# Extirpation of the Kidney

**Published:** 1878-02

**Authors:** 


### Extirpation of the Kidney.

On Thursday, the 7th June, at the Leeds Infirmary, Mr. Jessop removed the left kidney from a child aged two years and three months. The first noteworthy symptoms were hæmaturia and irritation of the bladder, but several soundings for stone gave negative results. The child, however, lost flesh, and became more and more pallid. About two months ago a rapidly increasing tumour was discovered in the left renal region, and as the indications were those of malignant growth Mr. Jessop determined to cut down upon it, and, if possible to, remove it. The incision was similar to that recommended for colotomy, but longer. When the diseased mass was reached the kidney was peeled by means of the fingers, and a whip-cord ligature was passed around the vessels and ureter, and firmly tied. The remainder of the growth was afterwards stripped away, and the whipcord left to drain the wound. The operation was a formidable one owing to the large size of the diseased organ and the free venous hæmorrhage which followed the separation of the growth from the surrounding structures. When removed the kidney weighed sixteen ounces, and resembled encephaloid in appearance. The child was doing well on the 11th instant. There was no peritonitis, the bowels acted freely, and the urine flowed abundantly, and was not stained. There was no vomiting, the temperature was but little above normal, and the child partook freely of milk. Mr. Jessop has kindly promised to publish in due course a detailed account of the case. On going to press we learn that the symptoms in the case are all favorable.—*Lancet.*

———:o:———

An official report from the Indian Gevernment informs us that the great cyclone on the Bengal coast cost 165,000 lives per million of those who inhabited the district over which it extended.—*Medical News.*

